# Renal Infarction in a Kidney With Multiple Renal Arteries

**DOI:** 10.7759/cureus.73189

**Published:** 2024-11-07

**Authors:** Catarina Forra, Diana Brites, Olga Korobka, Sandra Martin, Maria Eugénia André

**Affiliations:** 1 Internal Medicine, Unidade Local de Saúde de Castelo Branco, Castelo Branco, PRT; 2 Internal Medicine, Unidade Local de Saúde da Cova da Beira, Covilhã, PRT

**Keywords:** anatomical variants, multiple renal arteries, renal artery infarction, renal vascular injury, vascular risk factors

## Abstract

Multiple renal arteries are common, and their damage can lead to ischemia in the segments they supply. Renal infarction is a rare condition with a nonspecific presentation, primarily caused by embolism from the heart. The association between these entities has only been described in renal transplant recipients. We present the case of a patient with renal infarction of an unknown etiology in a native kidney with multiple renal arteries, an association not previously described in the literature.

## Introduction

Multiple renal arteries are not uncommon, being more frequently unilateral and considered essential arteries [1]. Their damage can lead to ischemia in the segments they supply [2]. Renal infarction is a rare phenomenon caused by the complete or partial occlusion of the main renal artery or its segmental branches, leading to ischemia and renal necrosis. This is caused predominantly by embolism from the heart or more unusually from an in situ thrombosis [3,4]. The presentation is usually nonspecific, but the diagnosis should be considered in patients who develop sudden abdominal or flank pain and who do not have other explanations for their symptoms, such as urolithiasis or pyelonephritis. The treatment options include systemic or intravascular thrombolysis, surgery, anticoagulation, and antiplatelet therapy [3]. The actual incidence of renal infarction remains unknown [4]. The association between multiple renal arteries and renal infarction has only been described in renal transplant recipients [5]. We present a rare case of an idiopathic renal infarction in a kidney with multiple renal arteries.

## Case presentation

**Figure 1:**
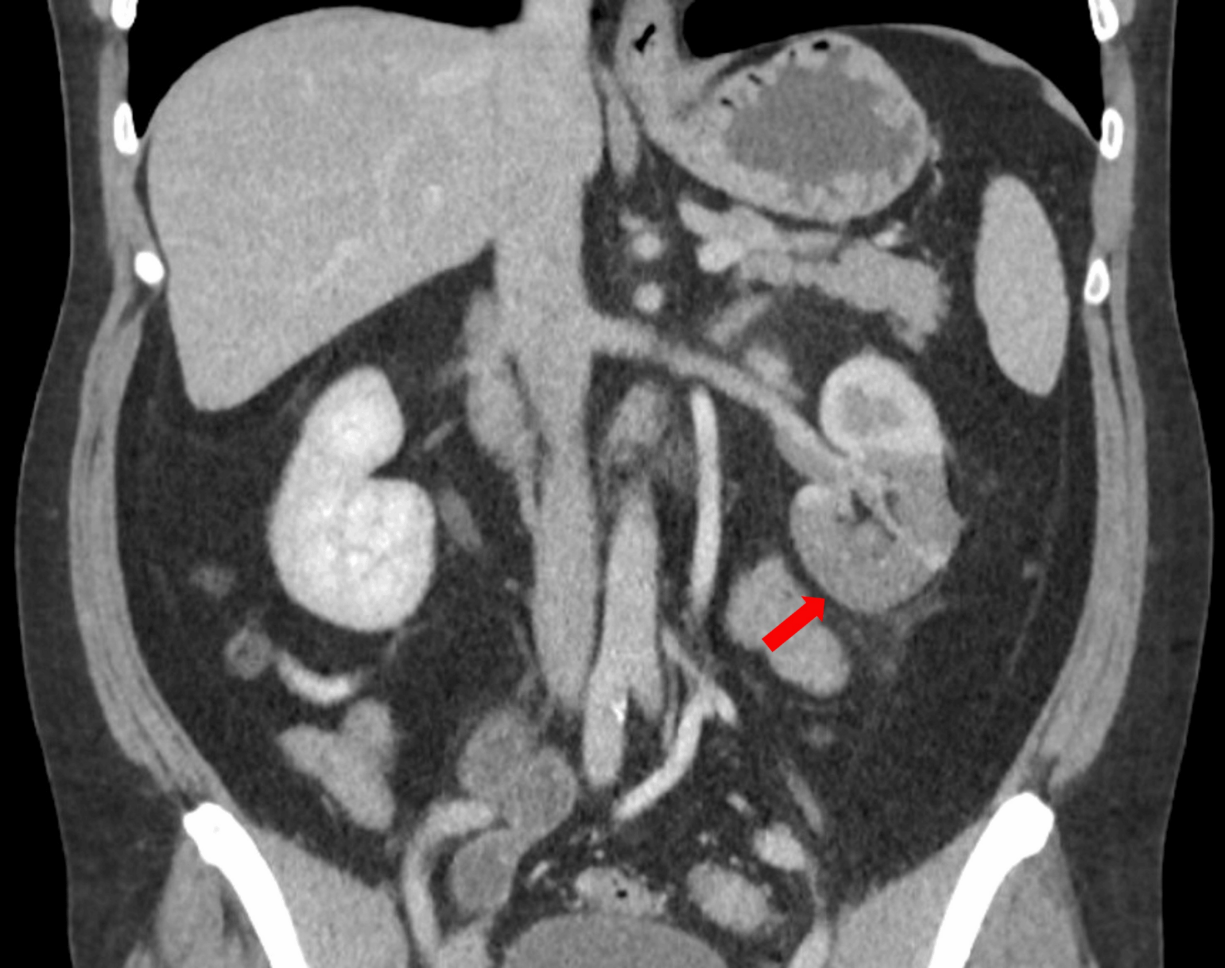
Computed tomography of the abdomen and pelvis revealing hypodense foci in the left kidney, suggesting parenchymal hypoperfusion in the medial and inferior portions (red arrow).

A male patient in his 30s presented to the emergency department (ED) with an eight-day history of intense left flank pain with no other associated symptoms. He had a previous history of active smoking and untreated hypertension. Upon arrival, the patient was in pain, afebrile, and with a blood pressure of 152/67 mmHg, a pulse rate of 80 beats per minute, and a room air saturation of 98%. The physical examination revealed sweaty skin and a normal abdominal palpation; other systemic examinations were unremarkable. Laboratory studies revealed mild leukocytosis (11,570/µL) and neutrophilia (6870/µL), with a negative C-reactive protein (CRP), normal renal function, normal lactate dehydrogenase (LDH), and unremarkable urinalysis. Due to a clinical suspicion of a renal colic, an abdominal and pelvic computed tomography (CT) was performed. The images revealed hypodense foci in the left kidney, suggesting parenchymal hypoperfusion in the medial and inferior portions, indicating renal infarction of an unknown etiology (Figure 1).

**Figure 2:**
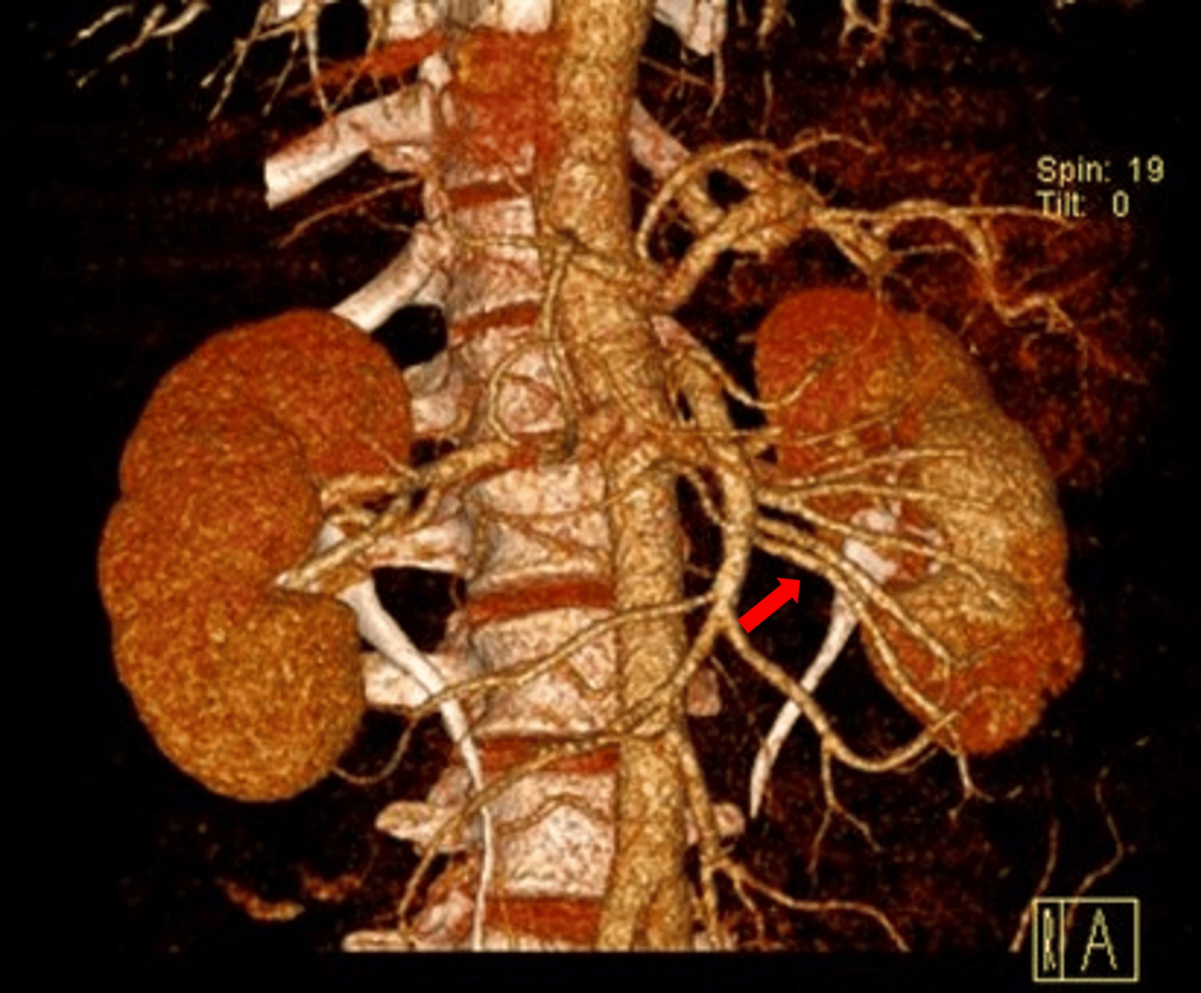
Computed tomography of the abdomen and pelvis with contrast (angiogram) revealing vascularization in the left kidney occurring through multiple renal arteries (red arrow).

After adding contrast and performing the angiogram, it was shown that the vascularization in the left kidney occurred through multiple renal arteries without any alterations in lumen patency (Figure 2).

After presenting the case to a vascular surgeon and urologist, who suggested anticoagulation and close monitoring, he was admitted for inpatient treatment and started on enoxaparin 1 mg/kg twice a day. During this period, he required opioids for pain control and antihypertensive agents. He was discharged one week later, improved, afebrile, and pain-free. From the etiological study, besides the known hypertension and active smoking, no other vascular risk factors were identified. Other workups, including echocardiogram, electrocardiography, thrombophilia, autoimmune, and coagulation studies, were normal.

One month later, he presented for a follow-up appointment, and the imaging revealed complete resolution of the infarction area. He remained on direct anticoagulants for three months, with continued clinical stability.

## Discussion

Flank pain is a common symptom encountered in the ED, but the diagnosis of renal infarction in this context is rare, with an incidence of under 1% in some studies [6,7], and more frequently identified postmortem. Although in recent years advances in imaging have led to a more frequent diagnosis of this condition [7], the underdiagnosis and delays in identification are primarily due to its nonspecific presentation, which mimics other conditions, particularly urolithiasis, but also pyelonephritis and other abdominal diseases [6], as observed in our patient.

The common symptoms usually include persistent abdominal or flank pain, which may or may not be associated with nausea, vomiting, hematuria, and fever [3,8]. Laboratory studies might reveal elevated serum levels of LDH, CRP, or hematuria [3,9]. Despite mild leukocytosis, our patient's blood work was within the normal range, adding to the nonspecificity of his complaints.

The causes of renal infarction include cardioembolic sources (the most common cause) [3,7], renal artery thrombosis, mechanical injury, aortic dissection, hypercoagulable states, and atheroembolic renal disease [3]. However, up to 30% of cases remain without an identifiable cause despite extensive workup [3,6]. In this patient, the major causes of renal infarction were excluded, although he had two identified vascular risk factors (active smoking and hypertension), which require aggressive management.

Among the cases of renal infarction, only in renal transplant recipients has the association between multiple renal arteries and the risk of infarction been described [5]. Multiple renal arteries are common, with studies showing an incidence ranging from 11% to 61%. CT is a method with high diagnostic value for both the morphological evaluation of the renal arteries [10] and the radiological diagnosis of renal infarction [8,11]. In the case presented, it was fundamental for diagnosing both conditions.

## Conclusions

To the best of our knowledge, the association between renal infarction and multiple renal arteries has not been previously described in the literature. While highlighting the importance of clinical suspicion of renal infarction in a patient with acute flank pain, the rarity of this case relates to the association between this vascular event and the presence of an anatomical variation of the renal arteries. We believe this warrants further studies to establish multiple renal arteries as a potential risk factor for renal infarction in native kidneys.
